# The impact of social–emotional competence on rural teachers’ intention to remain employed in China: a sequential mediation of teaching efficacy and job satisfaction

**DOI:** 10.3389/fpsyg.2026.1827908

**Published:** 2026-07-16

**Authors:** Chenfei Xiang, Tianping Wang, Jiachao Wei

**Affiliations:** 1 Faculty of Education, Southwest University, Chongqing, China; 2 School of Education Science, Nanning Normal University, Nanning, China

**Keywords:** job satisfaction, retention intention, rural teachers, social–emotional competence, teaching efficacy

## Abstract

**Introduction:**

Rural schools in China have long faced teacher shortages, high turnover, and workforce instability, which threaten instructional continuity and educational equity between urban and rural areas. Although policy initiatives have expanded the rural teacher supply, recruitment alone does not ensure long-term retention. This study examined how social–emotional competence (SEC), as an important psychological resource for teachers, is associated with rural teachers’ intention to remain employed (IRE).

**Methods:**

Survey data were collected from 861 rural teachers in China. A serial mediation model was used to examine whether teaching efficacy (TE) and job satisfaction (JS) mediated the relationship between SEC and IRE.

**Results:**

SEC had a significant total effect on IRE, but its direct effect became non-significant after TE and JS were included in the model. JS significantly mediated the relationship between SEC and IRE, and the serial mediation pathway SEC → TE → JS → IRE was also significant. However, the independent mediating effect of TE was not significant.

**Discussion:**

These findings indicate that SEC is associated with rural teachers’ retention intentions mainly through job satisfaction. Although TE alone does not directly translate SEC into stronger retention intentions, it functions as an upstream cognitive factor that supports retention indirectly by enhancing JS. The study clarifies an internal psychological pathway linking teachers’ individual resources, professional efficacy, affective experience, and retention intention. The findings suggest that efforts to retain rural teachers should not only improve external working conditions but also strengthen teachers’ SEC, professional efficacy, and job satisfaction, thereby promoting long-term commitment and workforce stability in rural schools.

## 1 Introduction

Rural education is a fundamental component of China’s education system and is central to advancing educational equity. Yet teacher shortages, high turnover and workforce instability have long constrained the development of rural schools, disrupting instructional continuity and further widening the urban–rural education gap. Teacher attrition is particularly acute in central and western China, where some village schools have reportedly been left with only one teacher (China News Network, 2017). Although the Special Post Teacher Programme has brought approximately 1.2 million teachers into rural classrooms over the past two decades (Ministry of Education of the People’s Republic of China, 2025), recruitment has not necessarily translated into sustained retention. According to the China Educational Statistics Yearbook, the number of rural teachers declined from 4.73 million in 2010 to 3.30 million in 2013 (Ministry of Education of the People’s Republic of China, 2013). Identifying the factors that shape rural teachers’ willingness to remain in the teaching profession is therefore crucial for stabilizing the rural teacher workforce and advancing educational equity (Chen and Li, 2021).

Existing research has primarily explained teacher retention through external or organizational factors, including salary, workload, professional development, school leadership and policy incentives. This body of work has provided an important foundation for understanding the structural challenges faced by rural teachers (Martin and Benedetti, 2025; Ingersoll, 2001; Ingersoll and Tran, 2023). However, such studies often conceptualize retention mainly as an outcome of external conditions, paying comparatively less attention to how rural teachers navigate demanding work environments through internal psychological mechanisms. A substantial body of research has also identified job satisfaction as a primary predictor of retention (Carver-Thomas and Darling-Hammond, 2017). Yet much of this literature stops at establishing a direct association between job satisfaction and retention, without further examining the core antecedents of job satisfaction or clarifying the complete psychological chain through which teachers’ internal characteristics progress from personal traits to cognitive appraisal, then to affective experience, and ultimately to retention-related decision-making.

Recent work has increasingly emphasized teachers’ personal psychological resources as potential determinants of retention. Among these resources, social–emotional competence (SEC) has been shown to be a key personal attribute that supports teachers’ professional persistence by helping them cope with multiple stressors and interpersonal challenges in rural teaching contexts. Teaching is inherently a form of emotional labour, requiring educators to regulate their own emotions while responding sensitively to students’ academic, social and emotional needs (Hong and Lee, 2016; Lee, 2019). In rural schools, these emotional demands may be especially pronounced because students often come from diverse and complex family backgrounds, family-based educational support is limited, and teachers are required to assume multiple roles and responsibilities. Prior studies have shown that higher SEC is associated with lower burnout and greater well-being (Goddard et al., 2004; Platsidou, 2010). However, existing research has yet to fully explain how SEC is translated into rural teachers’ intention to remain employed (IRE).

To address this gap, the present study adopts the Job Demands–Resources model (JD–R) as its overarching theoretical framework and conceptualizes SEC as a critical personal resource that enables rural teachers to manage high job demands under conditions of limited work resources (Bakker and Demerouti, 2007). Drawing further on conservation of resources theory and its emphasis on resource accumulation and resource gain (Halbesleben et al., 2014), this study proposes that SEC may first strengthen teachers’ positive cognition of their instructional capability, namely teaching efficacy, which may then enhance their affective evaluation of work experience, namely job satisfaction, and ultimately increase their intention to remain employed. Accordingly, this study constructs a serial mediation model linking social–emotional competence, teaching efficacy, job satisfaction and intention to remain employed, with a specific focus on the cognitive pathway, affective pathway and sequential process through which SEC shapes rural teachers’ retention intentions.

## 2 Literature review

### 2.1 Rural teachers’ intention to remain employed and SEC

Intention to remain employed refers to teachers’ willingness or psychological commitment to stay in their current profession or position. In teacher retention research, it is commonly regarded as an important antecedent of actual turnover behaviour, as teachers’ IRE reflects their evaluation of whether continued employment is sustainable at the personal, professional and emotional levels. For rural teachers, IRE is shaped not only by external conditions such as salary, workload, school management and professional development opportunities (Darling-Hammond, 2001; Montgomery and Rupp, 2005), but also by internal psychological resources that enable them to cope with stress and sustain professional engagement.

Social–emotional competence (SEC) refers to teachers’ capacity to understand and regulate their own emotions, identify students’ emotional needs, manage interpersonal relationships and make responsible decisions in educational settings. It is therefore likely to constitute a key internal psychological resource influencing teacher retention. Jennings and Greenberg (2009) argued that teachers with stronger social–emotional competence are better able to manage stress, build supportive teacher–student relationships and create positive classroom climates. Given the emotionally demanding nature of teaching, SEC affects not only the quality of teacher–student interactions, but also teachers’ ability to deal effectively with stress, conflict and negative emotions.

This capacity is especially important for rural teachers. Rural teachers often face inadequate instructional resources, limited family-based educational support, relatively weak professional support and multiple role responsibilities. Beyond classroom teaching, they may also need to address students’ emotional and behavioural difficulties, communicate with families, undertake administrative duties and provide care for students’ daily lives. Under such complex demands, teachers with higher SEC may be better able to regulate negative emotions, manage interpersonal challenges and maintain positive relationships, thereby reducing the psychological costs of teaching, sustaining professional engagement and developing stronger intentions to remain employed.

The Job Demands–Resources model posits that personal resources help individuals cope with job demands, reduce stress-related resource depletion and foster positive work attitudes. From this perspective, SEC can be understood as an important personal resource that enables rural teachers to respond to high job demands under conditions of limited external resources. Existing research has consistently shown that higher social–emotional competence is significantly associated with lower teacher burnout and greater occupational well-being (Goddard et al., 2004; Platsidou, 2010). These findings provide indirect evidence for a potential link between SEC and teachers’ intention to remain employed.

### 2.2 The cognitive pathway: teaching efficacy

Teaching efficacy refers to teachers’ belief in their ability to organize and implement instructional activities that promote desired student outcomes (Bandura, 1997). It represents a cognitive judgement of professional capability. For rural teachers, this belief is particularly important because they are often required to address instructional challenges, resource constraints and complex student needs under conditions of relatively limited external support. When teachers believe that they can manage classrooms effectively, support students and achieve educational goals, they are more likely to develop professional resilience and sustain their engagement in teaching.

SEC may contribute to TE. Drawing on the Job Demands–Resources model, SEC can be regarded as a foundational personal resource that helps rural teachers cope with emotional labour, interpersonal interactions and complex instructional tasks. Personal resources may strengthen professional motivation by fostering positive perceptions of competence. In this sense, teaching efficacy may constitute a cognitive pathway through which SEC influences teachers’ intention to remain employed. Specifically, teachers with higher SEC are generally better able to regulate stress and negative emotions, recognize students’ social and emotional needs, manage classroom interactions appropriately and establish positive teacher–student relationships. These capacities may enhance the effectiveness of classroom management and instructional practices, generate more positive teaching experiences and, in turn, strengthen teachers’ beliefs in their own instructional capability.

This process is also consistent with social cognitive theory. Bandura (1997) emphasized that mastery experiences are a major source of self-efficacy. In rural schools, where teachers frequently encounter uncertainty and limited support, SEC may increase the likelihood of such mastery experiences by helping teachers maintain emotional control and build effective relationships with students.

Previous studies provide preliminary evidence for different links in this cognitive pathway. Platsidou (2010) found that trait emotional intelligence was significantly associated with teacher self-efficacy among special education teachers. Goddard et al. (2004) also highlighted the importance of teacher efficacy for teacher motivation and professional functioning. Chesnut and Burley’s (2015) meta-analysis further showed that TE is closely related to teacher persistence and lower turnover intention. Together, these findings suggest that SEC may influence IRE through the cognitive mechanism of TE. Based on the above theoretical reasoning, this study proposes the following hypothesis.

*H1*: Teaching efficacy mediates the relationship between Social-Emotional Competence and Intention to Remain Employed among rural teachers.

### 2.3 The affective pathway: job satisfaction

In addition to cognitive beliefs about professional capability, teachers’ retention intention is also shaped by their affective experience of work. Job satisfaction refers to teachers’ positive emotional evaluation of their work and professional role. Hur and Abner (2023) found that job satisfaction is an important predictor of employee retention across organizational settings. In rural schools, job satisfaction may be particularly important because external rewards, such as salary, career advancement opportunities, and access to urban amenities, are often limited. Under such circumstances, teachers’ emotional attachment to teaching and their sense of fulfillment may become important reasons for remaining in their positions.

SEC may serve as an antecedent of job satisfaction because it enables teachers to manage emotional pressure, reduce interpersonal conflict, and develop supportive relationships with students and colleagues. Teachers with stronger SEC are more likely to interpret daily teaching challenges constructively and derive meaning from positive classroom interactions. In rural schools, where professional isolation is common, the relationship-management component of SEC may be especially valuable because it helps teachers build emotional support and reduce feelings of loneliness.

Previous research supports the association between teachers’ social–emotional competence and job satisfaction. Price’s causal model of voluntary turnover further suggests that turnover-related decisions are shaped through multiple exogenous and intervening factors, among which job satisfaction plays a central role in linking work conditions and individual experiences to retention-related intentions (Price, 2001). Vrnik Pere et al. (2020) demonstrated that teachers’ social and emotional competencies are related to a positive work environment and higher job satisfaction. Merida-Lopez et al. (2022) further found that job satisfaction mediates the relationship between teachers’ emotional competence and intention to quit. These findings suggest that job satisfaction may function as an affective pathway through which SEC influences retention-related outcomes.

Nevertheless, prior studies have not sufficiently examined this affective pathway in the context of rural teachers in China. Existing research often focuses on general teacher populations or examines emotional competence mainly in relation to burnout and well-being. The present study contributes by investigating whether job satisfaction explains the relationship between SEC and IRE in a rural educational context where intrinsic satisfaction may be especially important for sustaining teachers’ professional commitment.

*H2*: Job satisfaction mediates the relationship between social-emotional competence and rural teachers’ intention to remain employed.

### 2.4 The sequential mediating roles of TE and job satisfaction

TE and job satisfaction are conceptually distinct but theoretically connected. TE represents teachers’ cognitive belief that they are capable of achieving desired instructional outcomes, whereas job satisfaction reflects teachers’ affective evaluation of their work. This distinction is important because rural teachers’ intention to remain in their positions may depend not only on whether they believe they can teach effectively, but also on whether this belief is transformed into emotional fulfillment and satisfaction with their work.

The relationship between TE and job satisfaction can be understood as a process from professional capability beliefs to affective work experience. When teachers believe that they can manage classrooms, support students, and achieve instructional goals, they are more likely to experience accomplishment, professional meaning, and emotional reward. These positive experiences may then enhance job satisfaction. The two-factor theory provides a theoretical basis for this logic by identifying achievement and recognition as intrinsic motivators of job satisfaction (Herzberg et al., 1959). Similarly, Skaalvik and Skaalvik (2011) found that teachers’ sense of accomplishment and self-efficacy are related to job satisfaction and motivation to remain in teaching.

This sequential relationship is particularly relevant in rural schools. SEC may first help teachers regulate emotions, handle classroom challenges, and maintain positive relationships with students. These experiences may strengthen TE by increasing teachers’ belief in their professional capability. However, a belief in teaching capability alone may not be sufficient to sustain IRE unless it is accompanied by positive emotional experiences at work. Therefore, TE may further enhance job satisfaction by allowing teachers to experience achievement and fulfillment in daily teaching. Job satisfaction may then strengthen teachers’ intention to remain in rural schools.

This integrated model distinguishes the present study from previous research in three ways. First, it moves beyond studies that focus primarily on external predictors of rural teacher retention, such as salary, workload, and policy support. Second, it extends the literature on SEC by explaining how SEC may be converted into IRE through specific psychological mechanisms. Third, it integrates cognitive and affective mechanisms into a sequential mediation framework, suggesting that SEC may first strengthen teachers’ professional capability beliefs, which may then enhance their emotional satisfaction and ultimately support IRE. In this way, the present study provides a more cohesive theoretical explanation of how rural teachers’ internal psychological resources may contribute to professional commitment under resource-constrained conditions.

**Figure 1:**
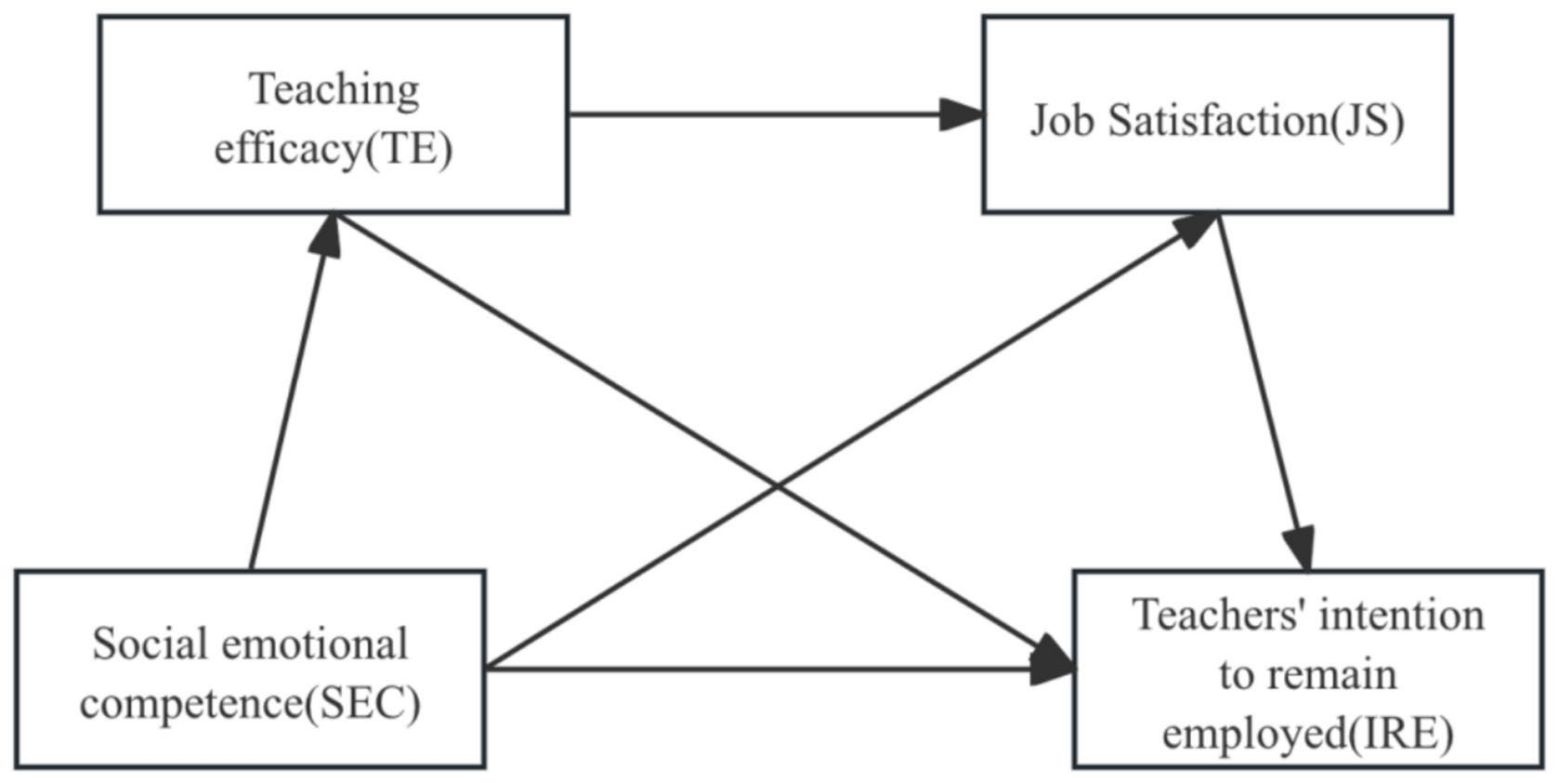
Conceptual framework.

*H3*: Teaching efficacy and job satisfaction sequentially mediate the relationship between social-emotional competence and rural teachers’ intention to remain employed (see Figure 1).

## 3 Method

This study adopted a quantitative, cross-sectional survey design to examine the relationships among rural teachers’ social–emotional competence, teaching efficacy, job satisfaction and intention to remain employed. A quantitative design was appropriate because the study aimed to test theoretically derived relationships among measurable variables and to examine direct and indirect effects within a mediation framework. Following Creswell’s (2014) view that research design should be selected in accordance with the research problem, research purpose and methodological procedures, the present study used standardized questionnaire data to assess the hypothesized relationships among the four constructs.

### 3.1 Participants

This study was conducted in County Z, a county-level rural education context in P Province, China. The name of the county has been anonymized to protect the confidentiality of the participating schools and teachers. However, sufficient contextual information is provided to clarify the sampling procedure and to allow readers to understand the empirical setting. County Z consists of 13 townships and 28 public rural schools. According to the 2023 local statistical bulletin, the total number of rural teachers in the county was 2,620.

County Z was selected as the research site for three reasons. First, it provides a typical county-level rural education context in which schools are distributed across both county-adjacent and remote township areas. Second, the county includes different types of rural schools, including junior high schools, primary schools, complete secondary schools, and nine-year compulsory education schools. Third, using one county-level context helped reduce broad regional differences in local policy, school administration, economic conditions, and cultural environment, allowing this study to focus more directly on the psychological mechanisms associated with IRE. A stratified sampling procedure was adopted based on the county’s geographic location, administrative zoning, township distribution, and the township affiliation of each school. The 13 townships and 28 schools were divided into four strata: the near-city core area, the northeastern area, the northern area, and the southern and southeastern ecological area. The near-city core area is geographically closest to the urban center and serves as the political, cultural, and educational core of the county; public schools in this area are relatively large in scale. The northeastern area is located in the northeastern part of the county, where educational resources are relatively evenly distributed and schools cover villages across the townships. The northern area is located in the northern part of the county, where townships are geographically connected and relatively concentrated; this area includes complete secondary schools, primary and secondary schools, and nine-year compulsory education schools. The southern and southeastern ecological area is the largest area of the county and mainly consists of remote townships; nine-year compulsory education schools are the dominant school type in this area.

The teacher population in the four strata was 939, 467, 587, and 627, respectively, totaling 2,620 rural teachers. The minimum required sample size was calculated using Cochran’s formula with a finite population correction. Based on a population size of 2,620 rural teachers, a 95% confidence level, an estimated proportion of 0.50, and a margin of error of 3.5% (Cochran, 1977), the minimum required number of valid responses was approximately 604.

Because questionnaire surveys may involve non-response and invalid responses, this study adopted a conservative expected valid response rate of approximately 60% to ensure that the final number of valid responses would remain above the required threshold. Accordingly, at least 1,007 questionnaires were required to obtain the minimum number of valid responses. A total of 1,008 questionnaires were therefore distributed proportionally across the four strata, following the teacher population distribution in each stratum. This procedure was also consistent with the need to account for potential non-response in survey research (Baruch and Holtom, 2008).

After excluding incomplete and invalid responses, 861 valid responses were retained, yielding a valid response rate of 85.4%. The final valid sample exceeded the minimum required sample size and provided an adequate sample size for the subsequent mediation analysis (Fritz and MacKinnon, 2007).

All participants were full-time rural teachers working in junior high schools, township primary schools, or village schools in County Z. Participation was voluntary, and respondents were informed that their answers would be used only for academic research. No personally identifiable information was collected. The questionnaire allocation across strata is reported in Table 1.

**Table 1:** Distribution of rural teacher population and questionnaire allocation.

Stratum	Teacher population	Population proportion	Questionnaires distributed
Near-city core area	939	35.8%	361
Northeastern area	467	17.8%	180
Northern area	587	22.4%	226
Southern and southeastern ecological area	627	23.9%	241
Total	2,620	100%	1,008

### 3.2 Research instruments

Rural teachers’ social–emotional competence was assessed using a 22-item scale developed by Li et al. (2021), an instrument that adapts the foundational CASEL (2003) framework to the Chinese cultural context to assess teachers’ social–emotional competence in Chinese educational contexts (Du and Mao, 2018). Teaching efficacy was measured using an instrument adapted from the Teacher Efficacy Scale (Gibson and Dembo, 1984) and further localized for Chinese educators by Yu et al. (1995). Job satisfaction was evaluated based on the Teacher Job Satisfaction Questionnaire developed by Xu and Zhao (2012). Finally, IRE was assessed using a measurement tool adapted from Lin and Wang’s (2022) research on the retention of young rural teachers.

### 3.3 Statistical analyses

Data analysis was conducted in three sequential phases using SPSS 26.0 and the PROCESS macro. First, descriptive statistics and bivariate correlations were calculated. Second, the mediating effects of TE and job satisfaction were tested separately using the PROCESS macro for SPSS (Model 4) (Hayes, 2017). Third, the PROCESS macro (Model 6) was used to test the multiple mediation model.

In accordance with Hayes (2017) recommendations, the statistical significance of indirect effects was determined using the bias-corrected bootstrap procedure with 5,000 resamples. This approach is commonly recommended because it does not assume a normal distribution of the product of coefficients. Following standard criteria, an indirect effect is considered statistically significant if the 95% bias-corrected confidence interval (CI) does not include zero (Hayes, 2017).

## 4 Results

### 4.1 Common method bias test

Because all data in this study were collected through self-reported questionnaires from the same participants at a single point in time, there is a potential risk that common method bias (CMB) might artificially inflate the relationships between the measured variables. To assess this concern, the study employed Harman’s single-factor technique. An exploratory factor analysis was carried out on all measurement items from the four scales. The results revealed that seven factors with eigenvalues greater than 1 emerged, and the first unrotated factor accounted for 31% of the total variance, which is well below the widely accepted threshold of 40% (Podsakoff et al., 2003). Furthermore, a single-factor confirmatory factor analysis (CFA) was performed by loading all self-rated items onto one latent factor. The fit metrics of this single-factor model were poor, showing substantial deviation from acceptable fit thresholds *(χ2/df = 40.099, CFI = 0.396, GFI = 0.444, AGFI = 0.375, NFI = 0.390, and RMSEA = 0.166)*. Together, these results suggest that the study’s data and subsequent findings are not significantly compromised by common method bias.

### 4.2 Correlation analysis between variables

Descriptive statistics and bivariate correlations were calculated for SEC, TE, JS and IRE to provide an initial assessment of the relationships among the independent variable, mediators and outcome variable. This served to initially assess the proposed interconnections between the independent variable, mediators, and outcome variable. As detailed in Table 2, SEC was positively correlated with teaching efficacy (*r* = 0.306, *p* < 0.001), job satisfaction (*r* = 0.567, *p* < 0.001), and intention to remain employed (*r* = 0.210, *p* < 0.001). There was also a positive correlation between TE and both job satisfaction (*r* = 0.345, *p* < 0.001) and IRE (*r* = 0.094, *p* = 0.002). Furthermore, job satisfaction was positively correlated with the intention to remain employed (*r* = 0.373, *p* < 0.001). These findings preliminarily suggest a positive relationship between rural teachers’ SEC and their intention to remain in their positions.

**Table 2:** The descriptive statistics and correlations among latent variables.

Research variables	M	SD	1	2	3	4
1. SEC	4.061	0.596	1			
2. TE	3.188	0.612	0.306^***^	1		
3. JS	3.471	0.912	0.567^***^	0.345^***^	1	
4. IRE	3.124	1.158	0.210^***^	0.094^**^	0.373^***^	1

SEC, social–emotional competence; TE, teaching efficacy; JS, job satisfaction; IRE, teachers’ intention to remain employed. *N* = 861; *represents *p* ≤ 0.05, **represents *p* ≤ 0.01, ***represents *p* ≤ 0.001.

### 4.3 Sequential mediation analysis

To further examine the sequential mediation effects of TE and job satisfaction, Model 6 of the PROCESS macro was applied, with social–emotional competence as the independent variable and IRE as the dependent variable. Regression estimates for each path are reported in Table 3, while indirect, direct, and total effects are summarized in Table 4. As recommended by Hayes (2017), an indirect effect is considered statistically significant if the 95% bias-corrected confidence interval (CI), spanning from the lower limit (BootLLCI) to the upper limit (BootULCI), does not contain zero.

**Table 3:** Regression paths and standardized coefficients (*N* = 861).

Path	B	SE	t	*p*	Boot CI 95%		Standardized β
					Boot LLCI	Boot ULCI	
SEC → TE	0.314	0.033	9.411	0.000	0.249	0.380	0.306
SEC → JS	0.780	0.044	17.663	0.000	0.693	0.866	0.509
TE → JS	0.282	0.043	6.553	0.000	0.197	0.366	0.189
SEC → IRE	0.009	0.076	0.114	0.909	−0.140	0.157	0.004
TE → IRE	−0.075	0.065	−1.157	0.248	−0.201	0.052	−0.039
JS → IRE	0.487	0.050	9.738	0.000	0.389	0.585	0.384

**Table 4:** Mediation effects (*N* = 861).

Effect type/pathway	Effect	Boot SE	Boot CI 95%		Completely standardized effect
			Boot LLCI	Boot ULCI	
Total	0.408	0.065	0.281	0.535	0.210
Direct effect	0.009	0.076	−0.140	0.157	0.004
Ind1: SEC—TE—IRE	−0.023	0.021	−0.067	0.017	−0.012
Ind2: SEC—JS—IRE	0.380	0.045	0.295	0.471	0.195
Ind3: SEC—TE—JS—IRE	0.043	0.011	0.023	0.066	0.022
Total indirect effect(s)	0.399	0.051	0.301	0.502	0.205

First, the total effect of SEC on IRE was significant (Effect = 0.408, *t* = 6.291, *p* < 0.001), as shown in Table 4. This indicates that, before the mediators were included, SEC was positively associated with IRE.

After entering TE and JS into the model, SEC significantly predicted both TE (*B* = 0.314, *t* = 9.411, *p* < 0.001) and JS (*B* = 0.780, *t* = 17.663, *p* < 0.001), as shown in Table 3. Additionally, TE significantly predicted JS (*B* = 0.282, *t* = 6.553, *p* < 0.001), and JS significantly predicted IRE (*B* = 0.487, *SE* = 0.050, *t* = 9.738, *p* < 0.001) (see Table 3). However, neither SEC (*B* = 0.009, *p* = 0.909) nor TE (*B* = −0.075, *p* = 0.248) directly predicted IRE after accounting for the mediators (Table 3), suggesting a potential full mediation pattern.

This pattern was further supported by the bootstrap results in Table 4, where the total indirect effect of SEC on IRE was significant (Effect = 0.399, 95% CI [0.301, 0.502]). Because the confidence interval excludes zero and the direct effect was non-significant, the results are consistent with a full mediation pattern in this model.

Regarding the specific indirect pathways (Table 4), the indirect effect through TE alone (Ind1: SEC → TE → IRE) was non-significant (Effect = −0.023, 95% CI = [−0.067, 0.017]), indicating that TE does not mediate the SEC → IRE relationship by itself. However, the indirect effect through JS alone (Ind2: SEC → JS → IRE) was significant (Effect = 0.380, 95% CI [0.295, 0.471]). The sequential pathway involving both TE and JS (Ind3: SEC → TE → JS → IRE) was also significant (Effect = 0.043, 95% CI [0.023, 0.066]).

These results indicate that SEC was associated with higher job satisfaction both directly and indirectly through TE, and job satisfaction was subsequently associated with stronger IRE, supporting the sequential mediation model.

**Figure 2:**
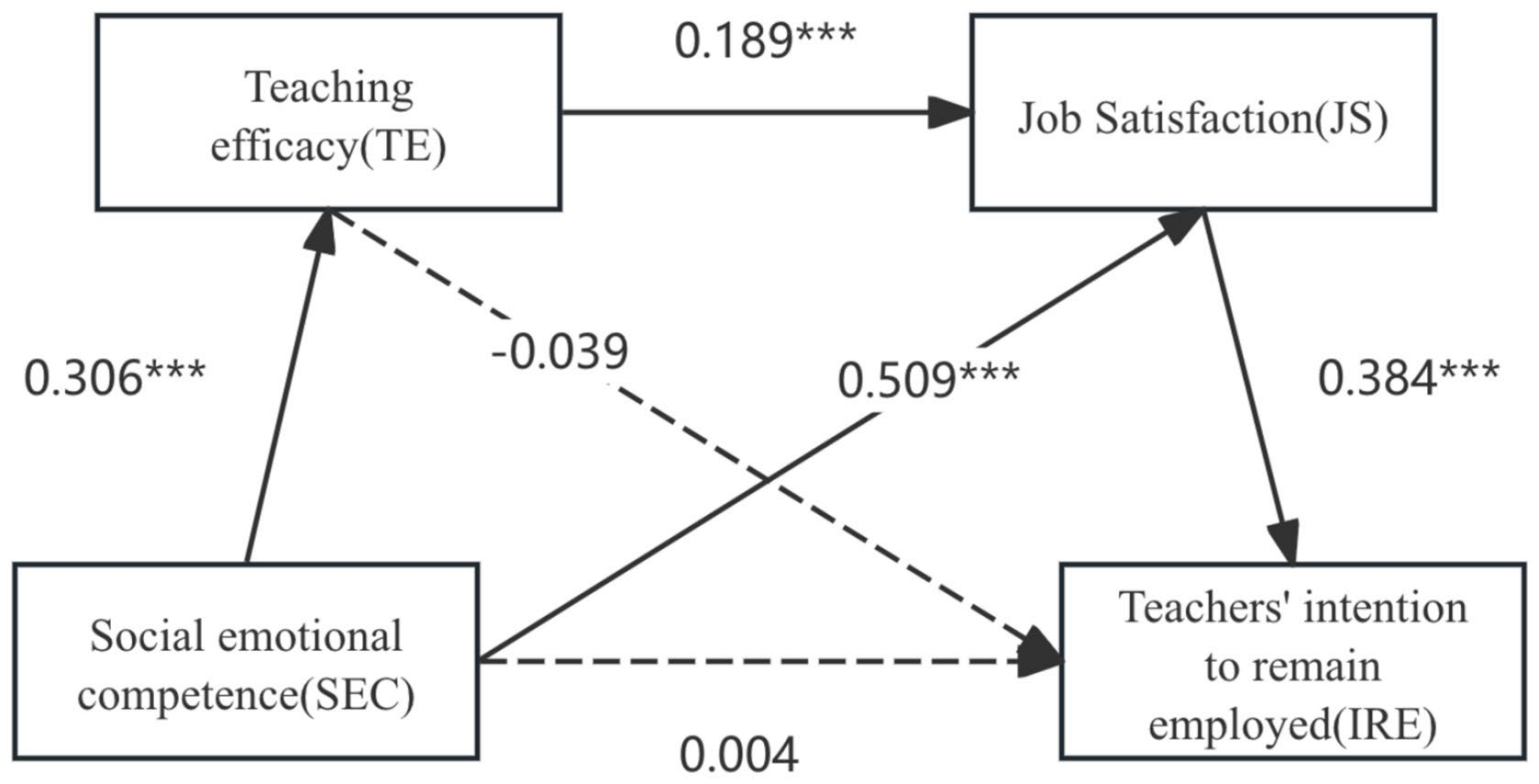
Sequential mediation model. Standardized coefficients (*β*) are presented. Solid lines indicate statistically significant paths, while dashed lines represent non-significant paths. ***p < 0.001.

Figure 2 displays the path diagram for the tested sequential mediation model, illustrating the standardized path coefficients (*β*) for all proposed relationships.

## 5 Discussion

### 5.1 Main findings

This study examined how social–emotional competence (SEC) is associated with rural teachers’ intention to remain employed (IRE) through teaching efficacy (TE) and job satisfaction (JS). Three main findings emerged from the sequential mediation analysis. First, the specific indirect effect of SEC on IRE through TE alone was not statistically significant, indicating that H1 was not supported. Second, JS significantly mediated the relationship between SEC and IRE, supporting H2. Third, the sequential indirect pathway SEC → TE → JS → IRE was statistically significant, supporting H3.

Taken together, these findings suggest that SEC is associated with rural teachers’ retention intention mainly through job satisfaction and, to a lesser extent, through a sequential cognitive-to-affective pathway in which teaching efficacy precedes job satisfaction. The results therefore do not support a simple interpretation that teaching efficacy alone explains why socially and emotionally competent teachers are more likely to remain in rural schools. Instead, they indicate that teaching efficacy becomes relevant to retention intention when it is connected to teachers’ affective evaluation of their work. In this sense, job satisfaction appears to be the more proximal mechanism linking SEC to IRE, whereas teaching efficacy functions as an upstream cognitive component within a broader psychological process.

### 5.2 Interpreting the differentiated mediation pattern

A central issue in interpreting the findings is the distinction between the non-significant simple mediation pathway and the significant sequential mediation pathway. As reported in Table 4, the specific indirect pathway SEC → TE → IRE was not statistically significant because its 95% confidence interval included zero. Therefore, H1 was not supported, and teaching efficacy should not be interpreted as a standalone mediator between SEC and IRE.

This finding indicates that, in the present model, teaching efficacy did not transmit the association between SEC and IRE by itself. Teaching efficacy reflects teachers’ beliefs about their professional capability, including their perceived ability to manage classrooms, support students, and respond to instructional challenges. These beliefs may be important for rural teachers, who often work with limited institutional support and complex student needs. However, the non-significant SEC → TE → IRE pathway suggests that perceived professional capability alone was not sufficient to form a significant indirect pathway from SEC to IRE.

The role of teaching efficacy becomes clearer when the significant sequential pathway is considered. Although TE did not function as an independent mediator, it was part of the significant pathway SEC → TE → JS → IRE. This result suggests that teaching efficacy is better understood as an upstream cognitive component whose relevance to retention intention operates through job satisfaction as a subsequent affective mechanism. In other words, the present findings support a sequential interpretation: SEC was associated with stronger teaching efficacy, teaching efficacy was associated with greater job satisfaction, and job satisfaction was associated with stronger intention to remain employed.

The role of job satisfaction was more direct. The significant indirect effect through JS indicates that job satisfaction served as a central affective mediator between SEC and IRE. This finding is consistent with previous research showing that teachers’ emotional competencies are related to job satisfaction and retention-related outcomes (Merida-Lopez et al., 2022; Vrnik Pere et al., 2020). It is also consistent with turnover and retention research suggesting that employees’ affective evaluations of their work are closely linked to their intention to remain in or leave an organization.

In rural schools, this affective pathway may be especially important. Rural teachers often face professional isolation, heavy responsibilities, limited promotion opportunities, and fewer material resources. Under such conditions, satisfaction derived from daily teaching may become an important source of professional commitment. Teachers with stronger SEC may be better able to regulate emotions, maintain constructive relationships, reduce interpersonal conflict, and derive meaning from classroom interactions. These experiences may support job satisfaction, which in turn is associated with stronger retention intention.

### 5.3 Implications of the sequential cognitive-to-affective pathway

The significant sequential mediation pathway provides a more nuanced explanation of how SEC is linked to rural teachers’ IRE. Specifically, SEC was associated with higher TE, TE was associated with higher JS, and JS was associated with stronger IRE. This pattern suggests a cognitive-to-affective process in which teachers’ social–emotional competence is first linked to their beliefs about professional capability and then to their affective evaluation of work.

This interpretation is consistent with Skaalvik and Skaalvik (2011), who showed that teachers’ sense of accomplishment and self-efficacy are closely related to job satisfaction and motivation to remain in teaching. In rural schools, teachers with stronger SEC may be better positioned to manage classroom relationships, respond empathetically to students, and cope with emotional demands. These experiences may strengthen their sense of teaching efficacy. However, the present findings suggest that efficacy beliefs appear to matter for retention intention mainly when they are linked to job satisfaction. Thus, the pathway from SEC to IRE is not simply cognitive; it depends on whether teachers’ professional capability beliefs are accompanied by positive affective experiences at work.

This pattern refines the application of the Job Demands–Resources (JD-R) model to rural teacher retention. Within the JD-R framework, SEC can be understood as a personal resource that helps teachers cope with high job demands and limited external resources. The present findings suggest that this personal resource is associated with retention intention through both an affective pathway and a sequential cognitive-to-affective pathway. Rather than operating through a single direct route, SEC appears to be linked to IRE through a process in which job satisfaction plays a central role and teaching efficacy contributes as an antecedent of that affective mechanism.

This interpretation also helps distinguish the present study from prior research on teacher retention. Previous studies have often examined external or organizational predictors, such as salary, workload, leadership, and policy support. The present findings shift attention to the internal psychological pathways through which teachers’ personal resources may be associated with retention intention. By showing that TE alone did not function as a standalone mediator, while JS and the TE–JS sequence were significant, the study provides a more mechanism-based account of rural teacher retention.

### 5.4 Practical implications for rural teacher retention

The findings have several practical implications for educational authorities, policymakers, and school leaders. First, professional development for rural teachers should not focus solely on pedagogical techniques. Because SEC was associated with both TE and JS, teacher development programs should include social–emotional learning components, such as emotion regulation, stress management, relationship building, and constructive communication. These components may help rural teachers develop the personal resources needed to cope with the emotional demands of rural teaching.

Second, the non-significant indirect effect through TE alone suggests that improving teachers’ professional efficacy may not be sufficient to strengthen retention intention. Training programs that focus only on instructional skills or confidence may have limited retention value if teachers do not also experience their work as satisfying and meaningful. School leaders should therefore create conditions that help teachers translate professional competence into positive work experiences. Such conditions may include regular recognition, constructive feedback, opportunities for professional autonomy, and meaningful participation in school decision-making.

Third, because JS emerged as the central affective mediator, rural teacher retention policies should give greater attention to job satisfaction. Educational administrators should reduce excessive administrative burdens and non-teaching tasks where possible, allowing teachers more time and energy for instructional work and student interaction. Schools should also foster supportive leadership, collegial collaboration, mentoring programs, and peer-support networks, particularly for young rural teachers. These measures may reduce professional isolation, strengthen teachers’ sense of belonging, and support their long-term willingness to remain in rural schools.

### 5.5 Limitations and future research

Several limitations should be acknowledged. First, this study used a cross-sectional design, which limits the ability to draw causal inferences about the ordering of SEC, TE, JS, and IRE. Although the tested model was theoretically grounded, the temporal sequence among these variables could not be confirmed with cross-sectional data. Future studies should employ longitudinal or experimental designs to examine whether changes in SEC precede changes in teaching efficacy, job satisfaction, and retention intention over time.

Second, the data were collected through self-reported questionnaires, which may introduce common method bias and social desirability bias. Although this study conducted statistical tests to assess common method bias, future research could strengthen methodological validity by using multi-informant or multi-source data. For example, teacher self-reports could be supplemented with peer evaluations, principal evaluations, classroom observations, or administrative retention records.

Third, this study treated SEC as an overall construct. Given the multidimensional nature of SEC, future research could examine its specific components, such as self-awareness, self-management, social awareness, relationship management, and responsible decision-making. Such analyses would help clarify whether different dimensions of SEC contribute differently to teaching efficacy, job satisfaction, and retention intention.

Finally, the study focused on rural teachers from one county-level context in China. Although this design allowed the study to examine rural teacher retention within a clearly defined educational setting, the generalizability of the findings may be limited. Future research could include rural teachers from different provinces, regions, and school types to test whether the proposed cognitive-to-affective pathway operates similarly across diverse rural educational contexts.

## 6 Conclusion

This study examined how SEC is associated with rural teachers’ intention to remain employed through TE and JS. The findings showed that JS served as a central affective mediator, while TE alone did not significantly mediate the SEC–IRE relationship. However, the significant sequential pathway SEC → TE → JS → IRE suggests that TE may function as an upstream cognitive component that becomes relevant through job satisfaction. These findings extend the JD-R perspective by identifying a cognitive-to-affective mechanism linking teachers’ personal resources to retention intention. Practically, the results suggest that rural teacher retention strategies should not focus only on recruitment or skill-based training, but should also foster teachers’ social–emotional competence, professional efficacy, and job satisfaction.

## Data Availability

The original contributions presented in the study are included in the article/supplementary material, further inquiries can be directed to the corresponding author.
